# Expression profile analysis of antisense long non-coding RNA identifies WDFY3-AS2 as a prognostic biomarker in diffuse glioma

**DOI:** 10.1186/s12935-018-0603-2

**Published:** 2018-07-28

**Authors:** Fan Wu, Zheng Zhao, Ruichao Chai, Yuqing Liu, Kuanyu Wang, Zhiliang Wang, Guanzhang Li, Ruoyu Huang, Haoyu Jiang, Kenan Zhang

**Affiliations:** 1 Department of Molecular Neuropathology, Beijing Neurosurgical Institute, Capital Medical University, Beijing, China; 2 Department of Neurosurgery, Beijing Tiantan Hospital, Capital Medical University, Beijing, China; 3 Chinese Glioma Genome Atlas Network (CGGA) and Asian Glioma Genome Atlas Network (AGGA), Beijing, China; 4 No. 6, Tiantan Xili, Dongcheng District, Beijing, 100050 China

**Keywords:** Glioma, LncRNAs, Antisense, Prognosis, Biomarker

## Abstract

**Background:**

Increasing evidence has shown that long non-coding RNAs (lncRNAs) are important prognostic biomarkers and epigenetic regulators with critical roles in cancer initiation and progression. However, the expression and clinical prognostic value of antisense lncRNAs in diffuse glioma patients remain unknown.

**Methods:**

Here, we profiled differentially expressed antisense lncRNAs in glioma using RNA sequencing data from Chinese Glioma Genome Atlas database. Cox regression was performed to evaluate the prognostic value. Gene oncology (GO) and gene set enrichment analysis (GSEA) were used for functional analysis of antisense LncRNAs.

**Results:**

Expression profiling identified 169 aberrantly expressed antisense lncRNAs between lower grade glioma (LGG) (grade II and III) and glioblastoma multiforme (GBM), 113 antisense lncRNAs between LGG IDH-wt and IDH-mut samples, and 70 antisense lncRNAs between GBM IDH-wt and IDH-mut samples, respectively. Among them, three antisense lncRNAs (WDFY3-AS2, MCM3AP-AS1 and LBX2-AS1) were significantly associated with prognosis and malignant progression of patients. WDFY3-AS2, the top one of downregulated antisense lncRNAs in GBM with fold change of 0.441 (*P *< 0.001), showed specific decreased expression in classical, mesenchymal, LGG IDH-wt, GBM IDH-wt or MGMT promoter unmethylated stratified patients. Chi square test found that WDFY3-AS2 was significantly associated with the clinical and molecular features of glioma. Univariate and multivariate Cox regression analysis indicated that WDFY3-AS2 was independently correlated with overall survival (OS) of patients. Kaplan–Meier analysis found that patients with high WDFY3-AS2 expression had longer OS than the low expression ones in the stratified cohorts. Additionally, GO and GSEA showed that gene sets correlated with WDFY3-AS2 expression were involved in regulation of synaptic transmission, glutamate receptor and TNF signaling pathway.

**Conclusion:**

Our findings provided convincing evidence that WDFY3-AS2 is a novel valuable prognostic biomarker for diffuse glioma.

**Electronic supplementary material:**

The online version of this article (10.1186/s12935-018-0603-2) contains supplementary material, which is available to authorized users.

## Background

Gliomas, the common primary brain tumor, mostly remain incurable, despite aggressive therapies with surgery, chemo and radiotherapy [1]. Glioblastoma multiforme (GBM) (IDH wild type) is the most aggressive glioma in adults characterized by glioma cell invasiveness and robust neovascularization, with a median survival of 15 months and 5-year survival rate less than 3% following standard treatment [2]. Due to the infiltrative growth and inherent resistance to chemo and radiotherapy, it is urgent to identify new targets to increase glioma patients’ survival. Although recent studies have found several genetic mutations and deregulated signaling pathways, these findings are still insufficient to uncover the molecular basis of gliomagenesis [3]. Since the coding genes only account for 1–2% of whole human genome [4], noncoding RNAs are emerging as new therapeutic targets in glioma.

Long non-coding RNAs (lncRNAs) are defined as transcripts longer than 200 nucleotides without protein-coding potential [5]. Antisense lncRNAs are a class of lncRNAs relative to their complementary protein-coding genes, which are orientated in an antisense direction [6]. Recent studies have demonstrated that antisense lncRNAs are involved in multiple biological processes, including cell proliferation, survival, migration, invasion and apoptosis through epigenetic modification and chromatin remodeling [7]. Moreover, accumulating reports found that dysregulated expression of antisense lncRNAs plays a crucial role in development and progression of various cancers. For example, it was reported that antisense lncRNA HAND2-AS1 was significantly down-regulated in endometrioid endometrial carcinoma, and HAND2-AS1 overexpression inhibited invasion and metastasis through inactivating neuromedin U [8]. Zhu et al. found that upregulation of lncRNA ZEB1-AS1 promoted tumor metastasis and predicted poor prognosis in hepatocellular carcinoma [9]. In glioma, Mineo et al. reported that lncRNA HIFA-AS2, upregulated in mesenchymal glioma stem cells, facilitated the maintenance of self-renewal in hypoxia niches [10]. Han et al. found that HOXA11-AS, a novel cell cycle-associated lncRNA, could serve as a biomarker and therapeutic target for glioma patients [11]. Our previous study reported that upregulation of lncRNA HOXA-AS3 promoted tumor progression and predicted poor prognosis in glioma [12]. Hence, understanding the role and prognostic value of cancer associated antisense lncRNAs would reveal new perspectives on the molecular basis of gliomagenesis.

In this study, we profiled differentially expressed antisense lncRNAs in diffuse glioma. Combined with prognosis and tumor progression, we identified a new antisense lncRNA WDFY3-AS2 with length of 3383 nt which is located in chromosome 4q21.23. WDFY3-AS2 downregulation was closely correlated with tumor grade and poor prognosis in patients. Bioinformatic analysis predicted that WDFY3-AS2 was involved in synaptic transmission, glutamate receptor and TNF signaling pathway. These results indicated that WDFY3-AS2 could be a potential prognostic biomarker for diffuse glioma.

## Methods

### Patients and tissues

309 diffuse glioma samples from the CGGA database were included in this study. Each sample was diagnosed by two neuropathologists according to the World Health Organization (WHO) classification guidelines of central nervous system tumors [13]. The study was approved by the ethics committee of Tiantan Hospital, and the written informed consent was obtained from all patients.

### Datasets

The RNA sequencing data and corresponding clinical information (age, gender, histology, methylguanine methyltransferase (MGMT) promoter status, isocitrate dehydrogenase (IDH) mutation status and 1p/19q status) were downloaded from CGGA database (http://www.cgga.org.cn) [14]. The characteristics of glioma patients are summarized in Table 1. IDH mutation and MGMT promoter status were determined by DNA pyrosequencing as described in previous study [15]. 1p/19q status was determined using dual-color fluorescence in situ hybridization. According to the standard quality control criteria, the raw RNA sequencing data was first processed to define improper reads which would be removed. Then, the gene expression was estimated using the reads per kilobase transcriptome per million reads (RPKM). The calculated gene expression data was further used to compare the expression difference among samples [15].

**Table 1:** Clinical characteristics of glioma patients

Characteristics	n
Age	
≤ 43	166
> 43	143
Gender	
Male	194
Female	115
Subtype	
Classical	69
Mesenchymal	65
Proneural	99
Neural	76
Grade	
II	104
III	67
IV	138
IDH	
mut	159
wt	150
MGMT promoter	
Methylated	136
Unmethylated	111
NA	62
1p/19q	
Non-codeleted	222
Codeleted	36
NA	51
LGG (II, III)	
IDH-wt	45
IDH-mut/1p/19q non-codeleted	81
IDH-mut/1p/19q codeleted	29
GBM (IV)	
IDH-wt	105
IDH-mut	33

*LGG* lower grade glioma, *IDH* isocitrate dehydrogenase,* MGMT* methylguanine methyltransferase

### Gene oncology (GO), Kyoto encyclopedia of genes and genomes (KEGG) and gene set enrichment analysis (GSEA)

GO was applied to analyze the main function of differentially expression genes. KEGG was performed to analyze the pathway enrichment (http://david.ncifcrf.gov/). GSEA was carried out to identify gene sets of statistical difference between two groups by using GSEA v3 software (http://www.brodinstitute.org/gsea/index.jsp) [16].

### Statistical analysis

Patients were divided into high-expression and low-expression groups based on WDFY3-AS2 level using the median value. Kaplan–Meier with 2-sided log-rank test was used to evaluate the OS differences between these two groups. Chi square test was performed to detect difference of the pathologic features between two groups of patients. Univariate and multivariate Cox regression analysis was conducted to identify independent prognostic factors. Significance analysis of microarray (SAM) was calculated the differential expression genes with R package “samr”. ROC curve analysis was used to predict OS with R package “pROC”. All statistical analyses were conducted using SPSS or R software. *P *< 0.05 was considered significant.

## Results

### Antisense lncRNAs profile in diffuse glioma using CGGA RNA sequencing data

**Fig. 1:**
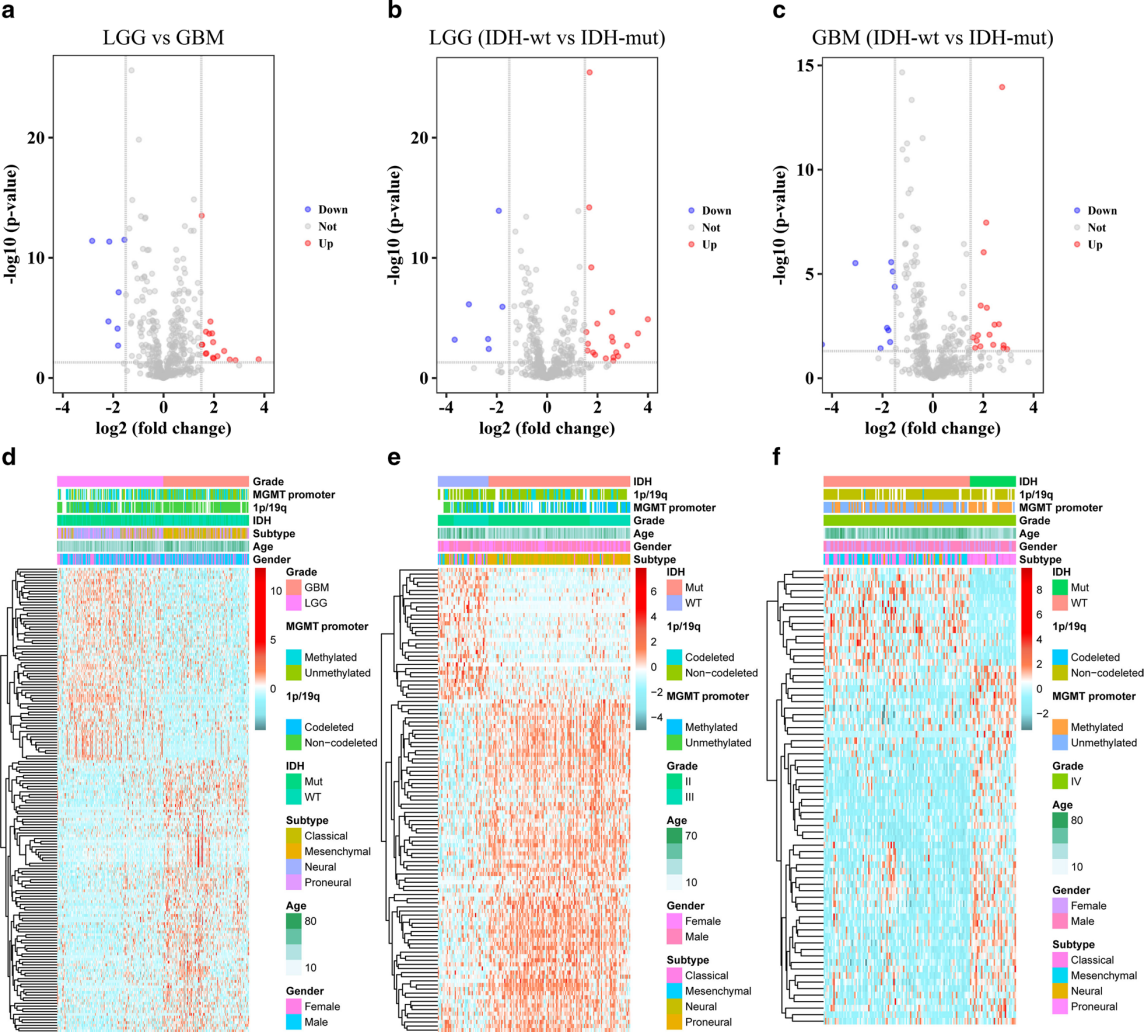
Expression analysis of antisense lncRNAs in diffuse glioma. **a**–**c** Volcano plots show the expression of 420 antisense lncRNAs between LGG and GBM, LGG IDH-mut and IDH-wt, GBM IDH-mut and IDH-wt samples. **d**–**f** Heat maps show the differential expression of antisense lncRNAs between LGG and GBM, LGG IDH-mut and IDH-wt, GBM IDH-mut and IDH-wt samples, respectively

The RNA sequencing data of 309 glioma samples was downloaded from CGGA database, and the clinicopathological features of the selected patients are listed in Table 1. Out of the whole transcriptome mRNAs, 420 antisense lncRNAs were identified. SAM with R package “samr” was performed to detect the differentially expressed antisense lncRNAs between LGG and GBM. Totally, 70 antisense lncRNAs were downregulated in GBM samples compared with LGG samples, while 99 antisense lncRNAs were upregulated in GBM samples. Volcano plot was used for showing the antisense lncRNAs of differential expression (Fig. 1a). Hierarchical clustering analysis conducted with R package “pheatmap” showed systematic variations in expression of these 169 antisense lncRNAs among samples (Fig. 1d). Considering the IDH mutation status, differentially expressed antisense lncRNAs within LGG and GBM samples stratified by IDH status were profiled. 113 antisense lncRNAs were abnormally expressed between LGG IDH-wt and IDH-mut samples (Fig. 1b, e), and 70 antisense lncRNAs were found between GBM IDH-wt and IDH-mut samples (Fig. 1c, f). These results suggested that the expression profiles of antisense lncRNAs were significantly different in lower grade gliomas and GBM.

### Screening of prognosis and tumor progression-related antisense lncRNAs

**Fig. 2:**
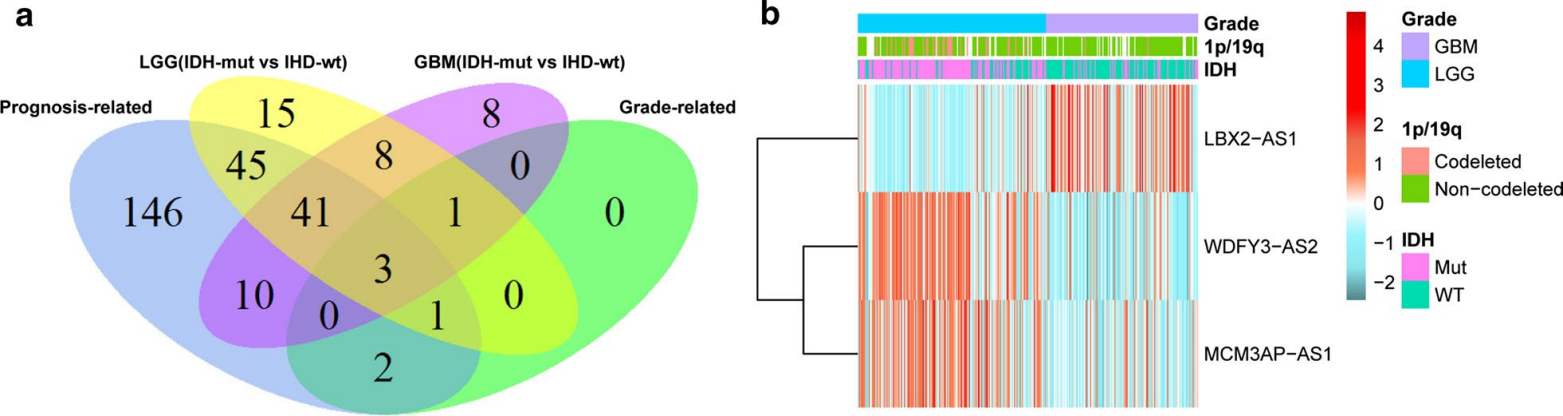
Screening of prognosis and tumor progression-related antisense lncRNAs. **a** Venn diagram presents the workflow used to identify antisense lncRNAs associated with prognosis and tumor progression. **b** Heat map shows the expression profile of three antisense lncRNAs (WDFY3-AS2, MCM3AP-AS1 and LBX2-AS1)

In order to find out antisense lncRNAs involved in prognosis and malignant progression in glioma, Cox regression analysis was performed using R package “survival”. Based on the Wald *P* values, 248 antisense lncRNAs were correlated with patients’ overall survival. Meanwhile, SAM was used to assess the differentially expressed antisense lncRNAs between tumor grades. Among samples of different grade, 47 (II vs III), 168 (II vs IV) and 55 (III vs IV) abnormally expressed lncRNAs were obtained respectively (*P *< 0.05), of which 6 overlapping antisense lncRNAs associated with grade progression were identified (Additional file 1: Figure S1). Combining prognosis, tumor progression and IDH status, three antisense lncRNAs were ultimately identified by computing the intersection. Among them, LBX2-AS1 was significantly upregulated, while the other 2 lncRNAs (WDFY3-AS2 and MCM3AP-AS1) were significantly downregulated as tumor grade increased. The Venn diagram presented the workflow used to identify antisense lncRNAs associated with prognosis and tumor progression (Fig. 2a). Heat map showed the expression profile of these three antisense lncRNAs (Fig. 2b).

### WDFY3-AS2 preferred downregulation in malignant glioma

**Fig. 3:**
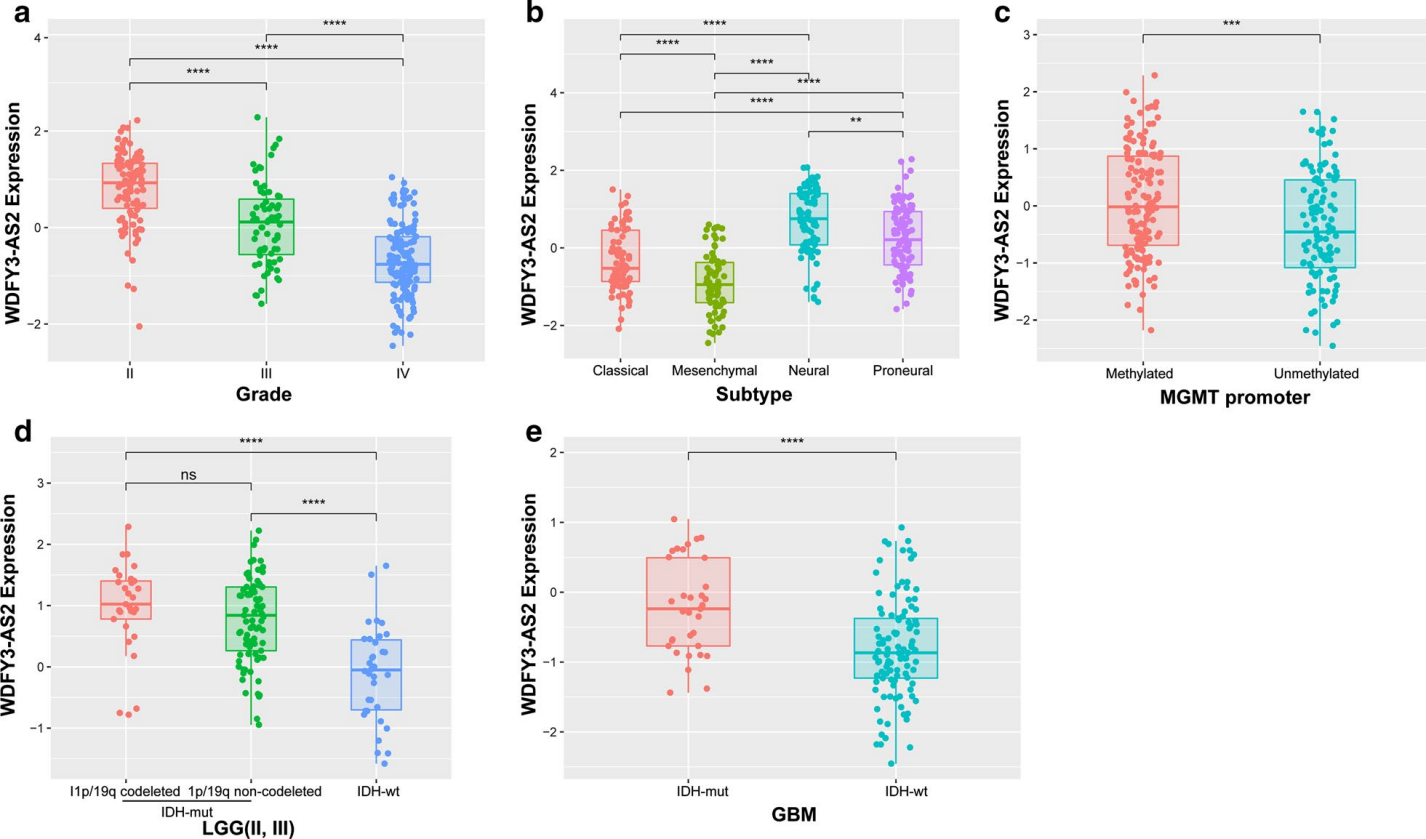
WDFY3-AS2 expression in stratified patients. **a** WDFY3-AS2 expression among different pathologic grades. **b** WDFY3-AS2 expression among different subtypes of patients. **c** WDFY3-AS2 expression between MGMT promoter methylated and unmethylated patients. **d** WDFY3-AS2 expression between LGG IDH-mut and IDH-wt patients. **e** WDFY3-AS2 expression between GBM IDH-mut and IDH-wt patients

Among the obtained three antisense lncRNAs, WDFY3-AS2 was one of the most significantly downregulated lncRNAs in GBM with fold change of 0.441 (*P *< 0.001). we further detected its expression in patients stratified by grade, subtype, IDH, 1p/19q and MGMT promoter status. As shown in Fig. 3, WDFY3-AS2 expression was downregulated along with histological grades, and the decreased expression was also observed in classical, mesenchymal, MGMT promoter unmethylated, LGG IDH-wt or GBM IDH-wt stratified patients. Additionally, the statistical difference of these pathologic features between high-expression and low-expression group of WDFY3-AS2 (based on the medial value) was assessed with Chi square test. Except gender (*P *= 0.102), most of features were found different between these two groups (Additional file 1: Table S1). These findings indicated a significant correlation between WDFY3-AS2 expression and clinical factors.

### WDFY3-AS2 showed strong prognostic power

**Fig. 4:**
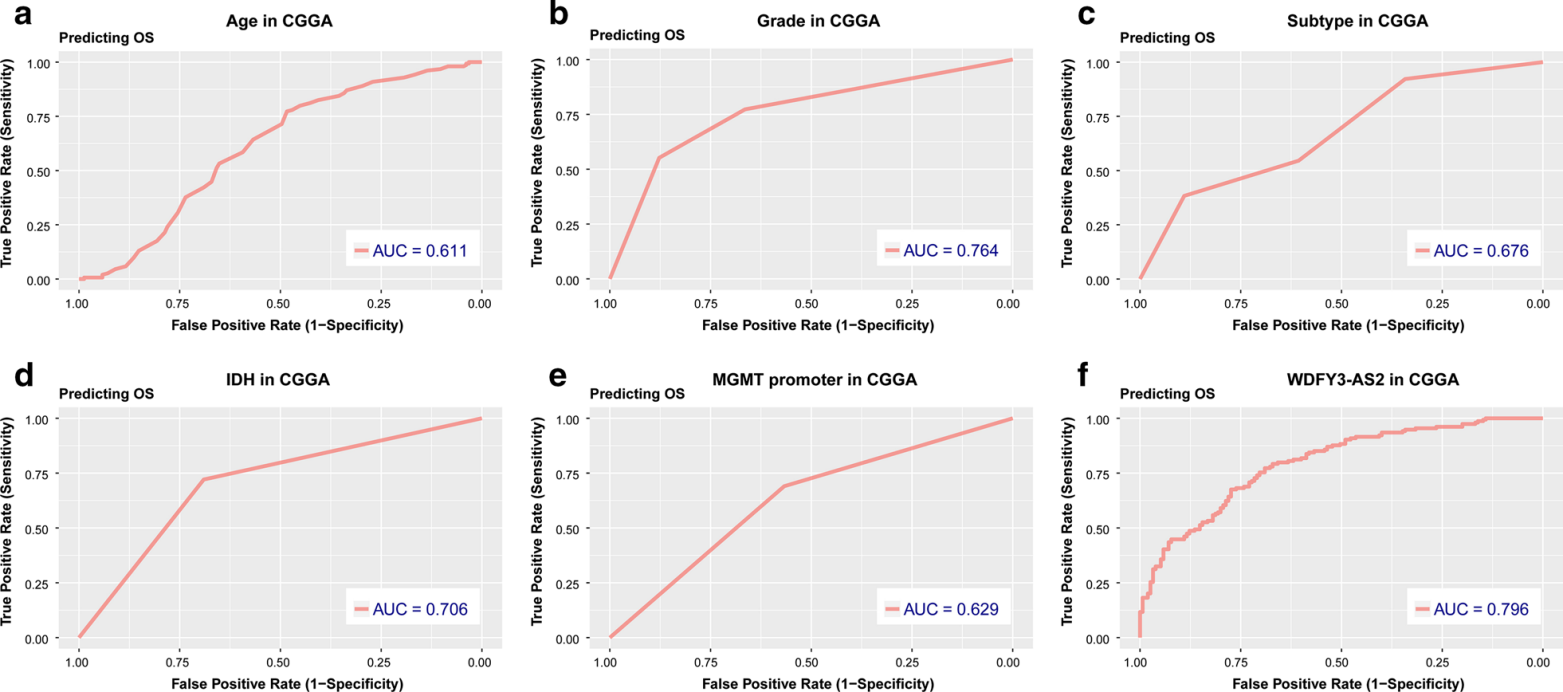
ROC analysis of age, grade, subtype, IDH, MGMT and WDFY3-AS2 for overall survival. *AUC* area under curve, *OS* overall survival

We further evaluated the prognostic value of WDFY3-AS2 for diffuse glioma patients. By using the expression of WDFY3-AS2 as a continuous covariate, univariate and multivariate Cox regression analysis were performed to determine the prognostic significance. The results showed that WDFY3-AS2 expression could serve as an independent prognostic factor in glioma patients (95% CI 0.423–0.771, *P *< 0.001) (Table 2). Furthermore, we assessed the predictive accuracy through computing the AUC (area under the curve) of WDFY3-AS2 and pathologic features with ROC curve. As shown in Fig. 4, the AUC of WDFY3-AS2 (0.796) was much higher than that of age (0.611), grade (0.764), subtype (0.676), IDH status (0.706) and MGMT promoter status (0.629). These data demonstrated the powerful ability of WDFY3-AS2 expression for predicting overall survival of diffuse glioma patients.

**Table 2:** Univariate and multivariate Cox regression analysis of clinical pathologic features for OS in CGGA cohort

Characteristics	Univariate analysis			Multivariate analysis		
	HR	95% CI	*P*-value	HR	95% CI	*P*-value
Age	1.038	1.022–1.053	<0.001	1.004	0.987–1.022	0.637
Gender	0.843	0.597–1.189	0.33			
Grade	3.469	2.709–4.443	<0.001	1.978	1.371–2.854	<0.001
Subtype	0.583	0.492–0.691	<0.001	0.869	0.711–1.062	0.17
IDH	0.229	0.159–0.331	<0.001	0.909	0.492–1.677	0.759
MGMT promoter	0.529	0.374–0.75	<0.001	0.773	0.511–1.169	0.222
1p/19q	0.165	0.067–0.404	<0.001	0.608	0.234–1.58	0.307
WDFY3-AS2	0.374	0.311–0.449	<0.001	0.571	0.423–0.771	<0.001

*HR* hazard ratio, *CI* confidence interval, *IDH* isocitrate dehydrogenase, *MGMT* methylguanine methyltransferase

### WDFY3-AS2 downregulation was associated with poor prognosis in diffuse glioma

**Fig. 5:**
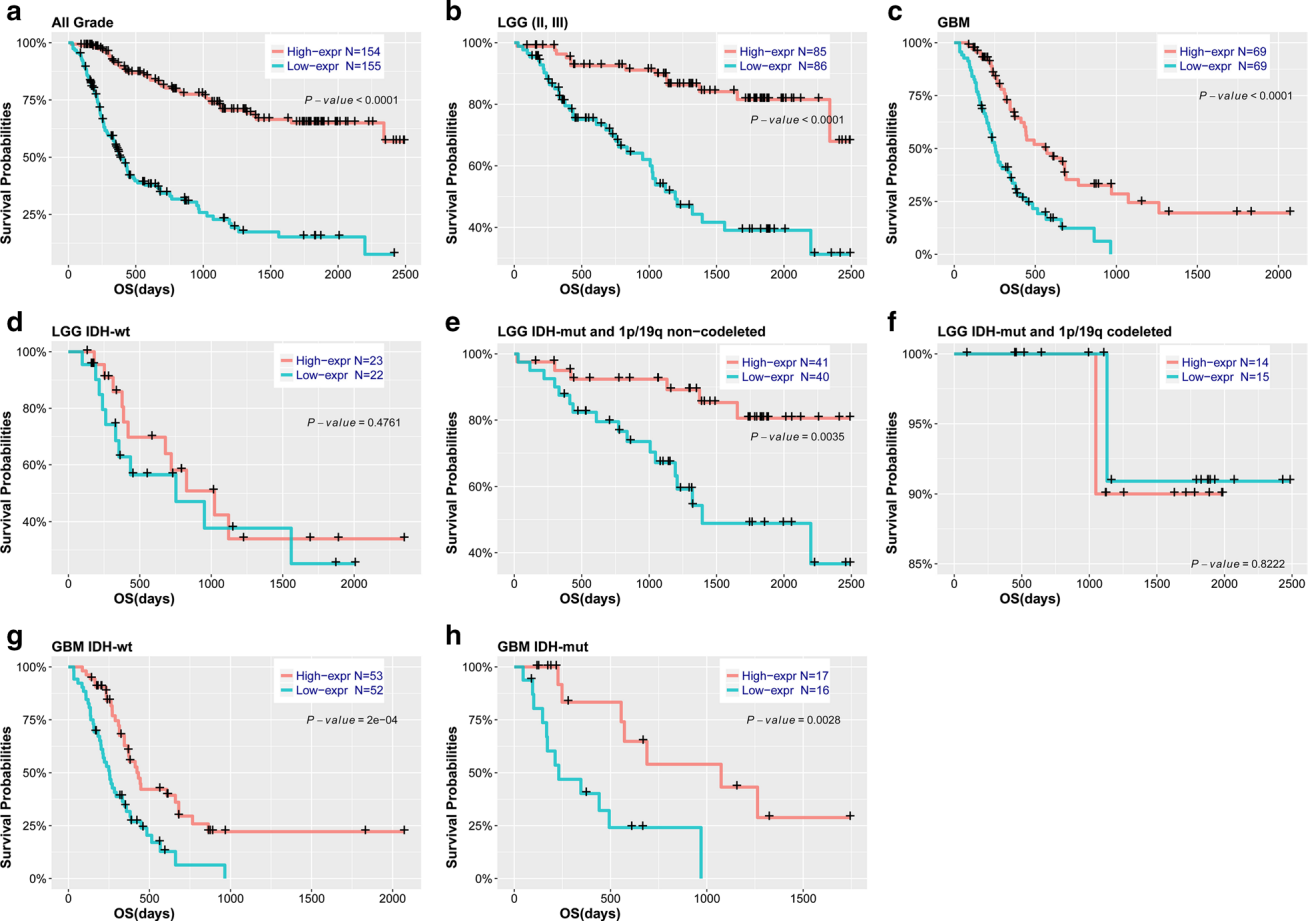
Survival analysis in patients stratified by grade, IDH and 1p/19q status based on WDFY3-AS2 expression. **a**–**c** Kaplan-Meier analysis of  WDFY3-AS2 in all grade, LGG and GBM patients. **d**–**f** Kaplan-Meier analysis of WDFY3-AS2 in LGG stratified by IDH and 1p/19q status. **g**, **h** Kaplan-Meier analysis of WDFY3-AS2 in GBM IDH-wt and IDH-mut patients

To further explore the relationship between WDFY3-AS2 expression and patients’ outcome, we also assessed the correlation between WDFY3-AS2 expression and overall survival. Based on the median value of WDFY3-AS2 expression, patients were divided into high expression and low expression groups. Kaplan–Meier analysis found that patients in low expression group had shorter OS than ones in high expression group (*P *< 0.001, Fig. 5a). Moreover, the similar trend was observed in patients stratified by grade, IDH status and 1p/19q status although no significant difference was found in IDH-wt, IDH-mut and 1p/19q codeleted patients (probably due to the small sample size) (Fig. 5b–h). Our results indicated that low level of WDFY3-AS2 was significantly associated with unfavorable prognosis in patients with diffuse glioma.

### Functional and pathway analysis of antisense lncRNA WDFY3-AS2

**Fig. 6:**
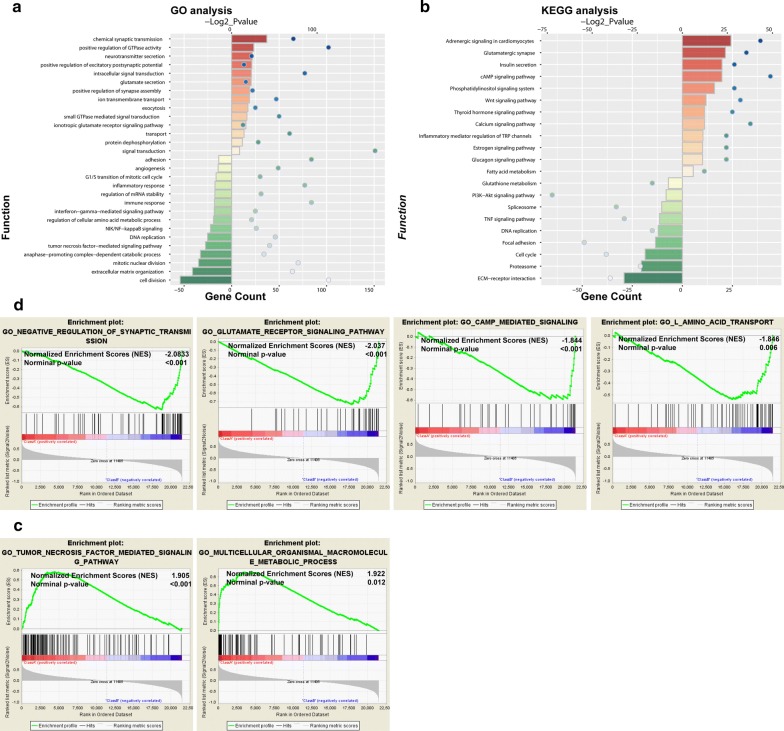
Functional and pathway analysis of antisense lncRNA WDFY3-AS2 in glioma. **a** GO annotations based on the top 2000 genes positively and negatively associated with WDFY3-AS2. **b** KEGG pathway analysis based on the top 2000 genes positively and negatively associated with WDFY3-AS2. **c**, **d** GSEA analysis based on the median value of WDFY3-AS2 expression level

To explore the function of WDFY3-AS2 in glioma, the differentially expressed genes between high and low expression groups of patients were identified by R package “samr”. GO and KEGG analysis were performed based on the top 2000 genes positively and negatively associated with WDFY3-AS2. GO enrichment found that the top involved biological processes were chemical synaptic transmission, positive regulation of GTPase activity, neurotransmitter secretion, cell division, extracellular matrix organization and mitotic nuclear division (Fig. 6a). Pathway analysis showed the top significant pathways included insulin secretion, cAMP signaling pathway, phosphatidylinositol signaling system, ECM-receptor interaction, proteasome and cell cycle (Fig. 6b). In addition, GSEA revealed that low expression of WDFY3-AS2 was associated with synaptic transmission, glutamate receptor signaling, cAMP mediated signaling and l-amino acid transport (Fig. 6c), while high expression was enriched in tumor necrosis factor mediated signaling and macromolecule metabolic processing (Fig. 6d).

## Discussion

Recently, large numbers of lncRNAs were found to be aberrantly expressed and involved in tumor initiation and progression in various cancers. Such lncRNAs could function as oncogenes and tumor suppressor genes and their expression could correlate with good or poor prognosis, making them valuable prognostic biomarkers. lncRNA HOTAIR has been used as a prognostic marker in different cancer types. High HOTAIR expression was significantly associated with poor overall survival and could serve as an independent prognostic factor [17]. It had been shown that MALAT1 was upregulated in many cancers and might act as a biomarker to predict survival in lung cancer [18]. lncRNA MEG3 was downregulated in a variety of primary cancers and found to be a prognostic factor for patients with glioma [19]. It was reported that lncRNA GAS5 expression was significantly reduced in liver cancer and could be an independent prognostic factor for patients [20]. In our study, we profiled differentially expressed antisense lncRNAs in glioma and identified a new antisense lncRNA WDFY3-AS2. Univariate and multivariate COX regression analysis found that WDFY3-AS2 downregulation was independently correlated with overall survival in patients, which indicated that WDFY3-AS2 could be a valuable prognostic biomarker for glioma.

As we known, increasing evidence has demonstrated that antisense lncRNAs are involved in regulation of a large range of biological processes through various mechanisms, such as transcriptional, post-transcriptional regulation and epigenetic modification. For example, antisense lncRNAs may regulate gene transcription in cis or trans through recruiting chromatin-modifying enzymes. Zhang et al. reported that lncRNA EZR-AS1 could enhance EZR transcription and expression by recruiting SMYD3 (a histone H3K4-methyltransferase) to its promoter region in esophageal squamous cell carcinoma [21]. lncRNA AGAP2-AS1 could bind with EZH2 and LSD1 and recruit them to KLF2 and LATS2 promoter regions to repress their transcription in non-small-cell lung cancer [22]. Antisense lncRNAs could function as competing endogenous RNAs (ceRNAs) to regulate targeted genes by binding with miRNAs. Wang et al. found lncRNA HOXD-AS1 promoted tumor progression by regulating the expression of frizzled family receptor 4 (FZD4) through competitively binding to miR-608 in ovarian cancer [23]. lncRNA TP73-As1 modulated cell proliferation through miR-200a dependent HMGB1/RAGE regulation in hepatocellular carcinoma [24]. In addition, antisense lncRNAs could also act as a molecular partner. Ding et al. reported that HNF1A-AS1 functioned as phosphatase activator through direct interaction with SHP-1 in hepatocellular carcinoma [25]. WDFY3, known as ALFY, encodes a phosphatidylinositol 3-phosphate-binding protein that function as a master conductor for aggregate clearance by autophagy [26]. Tschan reported that WDFY3 is critical for the granulocytic differentiation of AML cells [27]. Since antisense lncRNAs generally function as regulators of their complementary protein-coding genes [21], we first detected the expression correlation between WDFY3-AS2 and WDFY3 to evaluate the possible function and mechanism of WDFY3-AS2 in glioma. As shown in Additional file 1: Figure S1, WDFY3-AS2 expression was positively correlated with WDFY3 level in glioma (Pearson correlation coefficient r^2^ = 0.6418, *P *< 0.001), indicating a possible regulatory relationship between WDFY3-AS2 and WDFY3. Moreover, we also predicted the miRNAs that may interact with WDFY3-AS2 using LncBase v2 (https://omictools.com/lncrna2target-tool). mir-221, mir-26a, mir-135a, mir-9 and mir-139 showed the potential interaction with WDFY3-AS2. Among these miRNAs, mir-221 and mir-26a were overexpressed and could promote the malignant progression in glioma [28–30]. Therefore, we speculated that WDFY3-AS2 might modulate glioma cell malignant phenotype through interacting with mir-221 or mir-26a. GO and GSEA revealed that gene sets correlated with WDFY-AS2 expression were involved in regulation of synaptic transmission, glutamate receptor and TNF signaling pathways. Glutamate receptor signaling pathway plays important role in regulating glioma cell proliferation and invasion [31, 32]. Ciceroni found that type-3 glutamate receptors regulate chemoresistance in glioma stem cells, and their levels are inversely related to survival in patients with gliomas [33]. TNF signaling pathway is also an important regulator of the progression of glioma. Glioma cells tend to develop a resistance mechanism opposing to the TRAIL-induced apoptosis by overexpressing a wide variety of antiapoptotic proteins [34]. Deshayes reported that abnormal production of the TNF-homologue APRIL increases the proliferation of human malignant glioblastoma cell lines via a specific receptor [35]. Therefore, we proposed to determine whether WDFY-AS2 affected glioma cell behaviors through regulating glutamate receptor or TNF signaling pathway. Overall, further studies are required to clarify the function and molecular mechanism of WDFY3-AS2 in glioma.

## Conclusion

In summary, we profiled abnormally expressed antisense lncRNAs in diffuse glioma and identified a new antisense lncRNA WDFY3-AS2 whose expression was closely correlated with tumor grade and poor prognosis in patients. Bioinformatic analysis predicted that WDFY3-AS2 was involved in synaptic transmission, glutamate receptor and TNF signaling pathway. Our data suggested that WDFY3-AS2 might be a potential prognostic biomarker for diffuse glioma.

## Additional file

**Additional file 1.** Additional figures and table.
