# Supplementary material for: Expression profile analysis of antisense long non-coding RNA identifies WDFY3-AS2 as a prognostic biomarker in diffuse glioma
Source: Cancer Cell Int. 2018 Jul 28;18:107. doi: 10.1186/s12935-018-0603-2 (PMC6064140; doi:10.1186/s12935-018-0603-2)

**Additional Table S1.** Correlation between WDFY-AS2 expression and clinicopathologic factors of glioma patients.

| **Characteristics** | **n** | **WDFY3-AS2** | | ***P*-value** |
| --- | --- | --- | --- | --- |
|  |  | **Low** | **High** |  |
| **Total Cases** | 309 | 155 | 154 |  |
| **Age** |  |  |  |  |
| ≤43 | 166 | 70 | 96 | **0.002** |
| >43 | 143 | 85 | 58 |  |
| **Gender** |  |  |  |  |
| Male | 194 | 107 | 87 | 0.102 |
| Female | 115 | 48 | 67 |  |
| **Subtype** |  |  |  |  |
| Classical | 69 | 46 | 23 | **<0.001** |
| Mesenchymal | 65 | 54 | 11 |  |
| Proneural | 99 | 39 | 60 |  |
| Neural | 76 | 16 | 60 |  |
| **Grade** |  |  |  |  |
| II | 104 | 14 | 90 | **<0.001** |
| III | 67 | 30 | 37 |  |
| IV | 138 | 111 | 27 |  |
| **IDH** |  |  |  |  |
| mut | 159 | 45 | 114 | **<0.001** |
| wt | 150 | 110 | 40 |  |
| **MGMT promoter** |  |  |  |  |
| Methylated | 136 | 67 | 69 | **0.043** |
| Unmethylated | 111 | 69 | 42 |  |
| NA | 62 | 19 | 43 |  |
| **1p/19q** |  |  |  |  |
| Non-codeleted | 222 | 114 | 108 | **<0.001** |
| Codeleted | 36 | 6 | 30 |  |
| NA | 51 | 34 | 17 |  |

IDH = isocitrate dehydrogenase; MGMT = methylguanine methyltransferase.

**Additional Figure S1.** Screening of tumor progression-related antisense lncRNAs. (A) Venn diagram presents the workflow used to identify antisense lncRNAs associated tumor progression. (B) Heat map shows the expression profile of 6 antisense lncRNAs (WDFY3-AS2, MCM3AP-AS1, INHBA-AS1, LBX2-AS1, FOXD2-AS1 and A1BG-AS1).

**
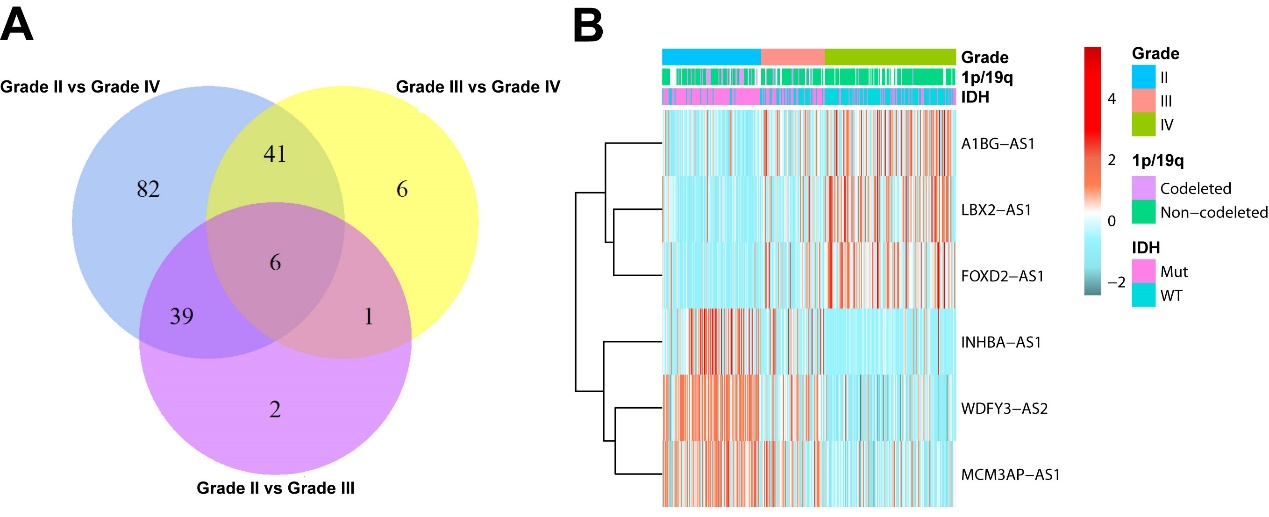
**

**Additional Figure S2.** The expression of WDFY3-AS2 was positively correlated with its complementary protein-coding gene-WDFY3. Correlation analysis between WDFY3-AS2 and WDFY3 expression. r^2^, Pearson correlation coefficient.


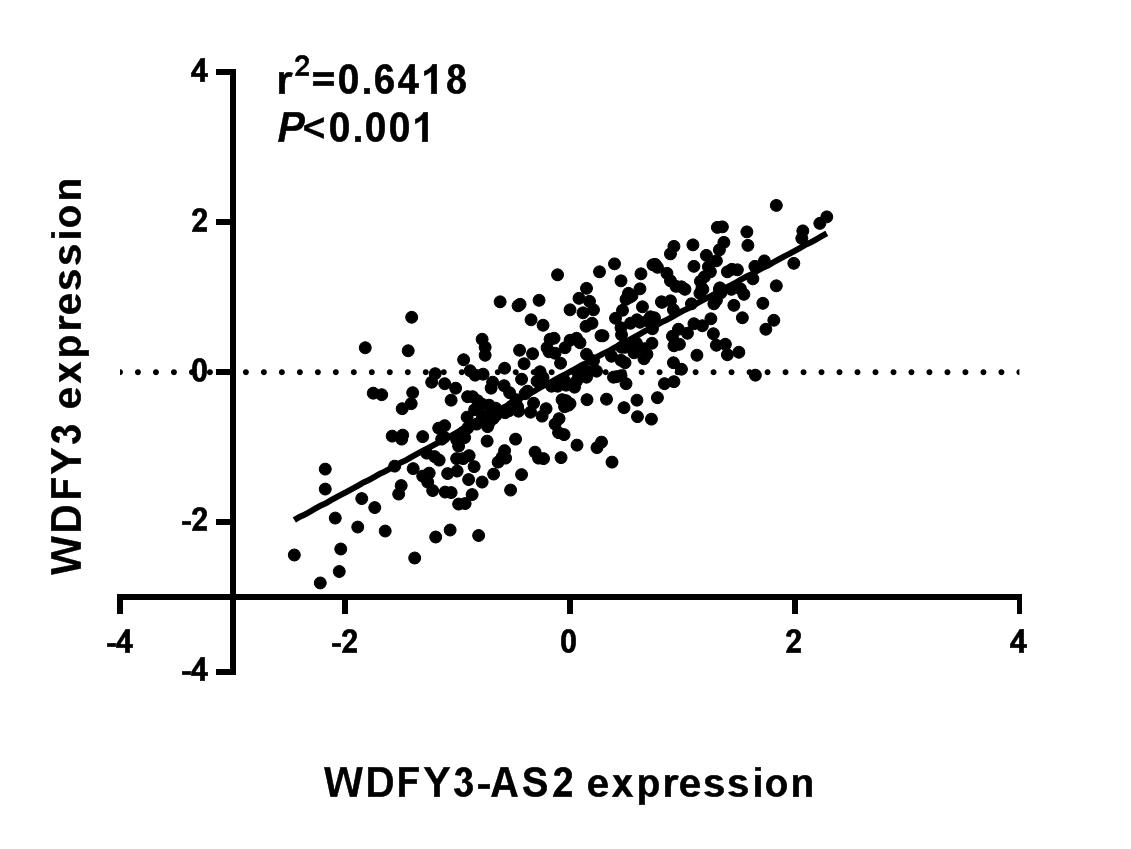

Supplement: Supplementary file 1 — Additional file 1. Additional figures and table. [file 12935_2018_603_MOESM1_ESM.docx]
